# Looking for a balance between visual and automatic sleep scoring

**DOI:** 10.1038/s41746-023-00915-7

**Published:** 2023-09-05

**Authors:** Vincenzo Muto, Christian Berthomier

**Affiliations:** 1 GIGA CRC In Vivo Imaging, Université de Liège, Liège, Belgium; 2 PHYSIP, Paris, France

**Keywords:** Biomedical engineering, Lab life, Sleep, Machine learning

## Abstract

Sleep recordings are visually classified in stages by experts in the field, based on consensus international criteria. This procedure is expensive and time-consuming. Automatic sleep scoring systems have, progressively over the years, demonstrated good levels of accuracy. Although the performance of these algorithms is believed to be high, however, there remains widespread skepticism in their daily use in clinical and scientific practice. In this comment to a recent publication of NPJ Digital Medicine, we express the reasons why we think the sleep expert should remain the central pivot in the pendulum between visual and automatic methodology, trying to find a new balance in the scientific debate.

Since the middle of the 20th century, sleep scoring has been defined as classification in sleep stages of 20–30s-epoch of an electroencephalogram (EEG) recording, based on international criteria^1^. These consensual rules, regularly revised by the American Academy of Sleep Medicine (AASM), enforce the choice of the used EEG derivations, and imply identification of specific graphoelements^2^ based on amplitude and frequency criteria. This staging classification is generally performed visually by sleep experts resulting in an expensive, in terms of human resources, and time-consuming process.

Like any method, the visual procedure has several limitations which affect its reliability, including inter- and intra-scoring variability, as for human experts, it is barely impossible to achieve the same classification for a given recording^3^, as well as maintaining consistency in the application of the scoring rules^4^. Moreover, the possible “drift” over time in visual sleep scoring represents a severe concern in clinical and epidemiological studies, speaking in favor of training sessions to ensure the homogeneous application of these rules^5^.

Consensual criteria interpret a biological process, based on an electrophysiological continuum, into a discrete categorization^6,7^. This discretization takes place at a double level, vertical (continuum of sleep states represented by the 4-stage sleep classification) and horizontal (time continuum summarized into 30-s scoring epoch), implying a distorsion in both dimensions and, therefore, an accepted loss of information. However, visual sleep scoring played an essential role in sleep science and still plays a pivotal role in research and clinical practice as it can detect unexpected events and thus reveal sleep abnormalities^8^.

Since the appearance of the first consensual criteria^1^ and the computers, a myriad of automatic sleep scoring methods have followed one another, growing in complexity in terms of method choice and hand in hand with scientific advances and technological resources. The latest method category that has been added is deep learning (DL). DL is a subset of machine learning techniques that use artificial neural networks to iteratively learn complex data-based representations by extracting features from the computing statistics, and establishing learning rules. DL-based algorithms do not require a priori knowledge about the physiological processes, the frequencies or amplitudes characterization or the kind of dataset used. They learn sleep features directly from the raw data.

While such approach free from priors is considered as a strength in many fields, the lack of interpretability and transparency, understood as inability to explain how decisions were made, gave rise to criticisms in the scientific community limiting its clinical acceptance^9,10^. In contrast, feature-based or rule-based algorithms are mainly based on a physiological ground that allows for understanding and interpretation of the criteria which can be then adapted or enriched by a human expert. This iterative approach involving a human expert seems more challenging in the deep learning method, as the expert cannot have direct access to the physiological meaning of the criteria. The criticism of DL algorithms as a black box is partially valid, as it is quite difficult to investigate which sleep features are encoded. Thus, the lack of explainability as well as the possibility of learning from biases inherent in the representativeness of the training databases, are weaknesses of the deep learning methods. From this prospective, explainable artificial intelligence (XAI) should help in building trust in the algorithms^11^. XAI can make artificial intelligence systems more explainable, by revealing more details of their inner workings. That said, there is no doubt, that the deep-learning approach can be extremely interesting in revealing new sleep signatures, but their relevance still needs to be validated by human experts.

Recently, in npj Digital Medicine, Fiorillo and colleagues used a DL algorithm previously validated by a different group^12^, in a large, heterogeneous multi-centers recordings sample, to assess how it would cope with the scoring task regardless of the EEG channel and the participants’ age^13^.

The authors assessed a fundamental issue: what is essential for visual scoring (specific EEG channels, specific scoring rules based on the age of the individuals) is not necessary for automatic analysis, which has other analysis principles than the eye of an expert.

The work illustrates that the rules of visual scoring, however, indispensable they may be in their objective of formalization and homogenization of sleep analysis, are above all an indication of how to process the recordings. This work also shows that there are alternatives, all the more so as the technological tools available for analysis are evolving.

The work of Fiorillo et al. supports the conclusions suggested by previously published methods that proposed to emancipate from the academic scoring rules (e.g., EOG based^14^, single EEG channel^15^, or motion based^16^), and offers the possibility of a reflexion on the topic of sleep automatic analysis and more generally on the complementary couple formed by science and technology.

From this point of view, the last 30 years have probably seen an excessive pendulum movement from no-automatic to all-automatic. For many years, there was a categorical refusal by the community to give credit to automatic analysis. It was true that the performance of autoscoring was sometimes uneven, but the question of visual analysis as the gold standard was hardly questioned. Algorithms were asked to reach 100% agreement with experts, which makes no sense given the inter-expert variability of 65–85%^17^. Thus, the discrepancies were systematically seen as errors from automatic methods.

Recently, on the contrary, there has been a huge craze for automatic analysis, as a result of the renewal of neural networks methods with the arrival of deep learning. Deep-learning techniques have generated a new enthusiasm and high expectations^18^. However, the comparison between algorithms performance is methodologically difficult because it raises the tricky question of a reference when comparing automatic vs. visual sleep scoring, and this question is not yet addressed in a homogeneous way throughout the literature. Some works that used the same methodology and reference showed that the improvement of performance between DL and non-DL methods is not really pronounced^9,19^.

In the face of this growing trend toward all-automatic, which suggests that visual analysis will eventually be replaced^8,20^, we want to emphasize that no matter how good the performance of autoscoring is, whether one uses DL or not DL, there will always be a need for an expert to access the raw data. There are two main reasons for this. First, if automatic analysis can indeed lighten the expert’s load in cases of a massive number of recordings in which it gives very good results (e.g., many cases of apnea), rare or complex pathologies (e.g., poly-medicated patients) require the expert’s eye. Secondly, the quality of raw data remains critical for the algorithms, which do not always have the means to detect signal anomalies (e.g., spurious or simply mislabeled signals, such as any non-EEG signal labeled as EEG). This risk exists in real life, and it may be difficult for an algorithm to detect these anomalies as it is not supposed to know a priori how degraded the signal may be. It may be tricky to distinguish, during a signal distortion, a physiological causality from a non-physiological one. These anomalies can lead to an erroneous automatic analysis, which an expert will be able to rule out.

Regardless of performance, much of the difficulty comes from the fact that autoscoring attempts to systematize a visual task that is not systematic but has much to do with expertise. So rather than trying to technically solve a poorly posed problem, let’s try to see how the technique can complement the existing investigation method for a researcher and a clinician, and contribute to reducing the current weaknesses of visual analysis, by decreasing inter- and intra-variabilities^21^ and make it possible to dig deeper.

Although the current sleep scoring system is primarily consensus-based rather than strictly scientific, the difference between scientific and technical aspects of sleep advances should be highlighted. Visual sleep scoring and signal processing are technical and methodological tools that allow us to understand the complex phenomenon of sleep, its content, and its meaning. Technology must serve science, not replace it. The development of automated sleep scoring algorithms is a key technical aspect of sleep understanding, but the responsibility for sleep scoring cannot be held solely by automated systems. Therefore, removing the expert from the sleep scoring process means losing the scientific aspect in favor of the technical one. As also pointed out by the authors, we have to imagine a hybrid solution, where the visual expert uses automatic tools as a way to perform faster and more in depth but without losing skills and expertise/specificity. We look forward to the development of sleep scoring algorithms based on artificial intelligence systems with explainable features that could reveal new consistent patterns across sleep recordings, and potentially help to better understand and manage signal degradation. This, in turn, could facilitate the revision of sleep scoring criteria.

In conclusion, we do need international consensus criteria and their regular updates but we don’t have to make them an absolute ground truth to be essentialized. Consensus rules are fundamental for a correct diagnosis as well as for the advancement of sleep science, because these rules represent the pivot on which science and medicine rest, as they guarantee the principle of reproducibility. However, these criteria should not prevent us from looking beyond the rules themselves when technical advances enable us to find new answers to clinical and scientific questions.
